# Exploring Psychiatric Home-Visit Nursing Practices for Patients with Schizophrenia and Hikikomori with a Thematic Analysis

**DOI:** 10.3390/ijerph21020181

**Published:** 2024-02-05

**Authors:** Maki Miou, Hirokazu Fujimoto, Kayano Yotsumoto, Misato Hirota, Satoshi Nishigaki, Takeshi Hashimoto

**Affiliations:** 1 Graduate School of Health Sciences, Kobe University, 7-10-2 Tomogaoka, Suma-ku, Kobe 6540142, Japan; yotumoto@kobe-u.ac.jp (K.Y.); nsmisato@people.kobe-u.ac.jp (M.H.); hashimo@kobe-u.ac.jp (T.H.); 2 Faculty of Nursing, Osaka Shin-Ai Gakuin University, 6-2-28 Tsurumi, Tsurumi-ku, Osaka 5380053, Japan; 3 Faculty of Nursing, Hyogo Medical University, 1-3-6 Minatojima, Chuo-ku, Kobe 6508530, Japan; 4 School of Nursing, Takarazuka University, 1-13-16 Shibata, Kita-ku, Osaka 5300012, Japan; s-nishigaki@takara-univ.ac.jp

**Keywords:** hikikomori, home-visit nursing, schizophrenia, nursing practice, qualitative research, thematic analysis

## Abstract

The phenomenon of some patients with schizophrenia withdrawing and becoming hikikomori needs to be resolved. In some countries, outreach methods are being employed. In Japan, psychiatric home-visit nursing for patients with schizophrenia and hikikomori is being implemented. However, it is not based on sufficient evidence and relies on the experience and intuition of individual nurses. This study explored the underlying themes in the nursing practices of psychiatric home-visit nurses via semi-structured interviews with 10 nurses and a thematic analysis. Nine key themes emerged. Four themes—(i) understanding the patient’s world, (ii) supporting the patients as they are, (iii) providing a sense of relief, and (iv) having equal relationships—highlighted the nurses’ commitment to respecting patients’ individuality while building and sustaining relationships. Two themes—(v) exploring the right timing and (vi) waiting for the appropriate timing—illustrated the nurses’ anticipation of proactive patient engagement. Finally, three themes—(vii) working together on things, (viii) continuing care for expanding the patient’s world, and (ix) nursing care for the patient’s future—underscored the nurses’ gradual and methodical approach to working alongside patients. Nursing practices based on these nine themes cultivated meaningful relationships and secured a sense of relief for the patients. Additionally, they awaited patients’ proactive engagement and delivered timely support to facilitate positive daily life changes. These findings contribute to the establishment of evidence-based nursing practices for patients with schizophrenia and hikikomori.

## 1. Introduction

Many patients with schizophrenia withdraw and become hikikomori. Schizophrenia is a specific syndrome consisting of positive symptoms, such as hallucinations and delusions, and negative symptoms, such as emotional flattering, decreased spontaneity, and social withdrawal [1]. Therefore, it is not uncommon for individuals with schizophrenia to retreat into a state of physical withdrawal owing to the influence of both positive and negative symptoms [2]. Additionally, patients with schizophrenia may opt for social isolation for reasons other than their symptoms, such as the avoidance of stressful events in community life [2].

“Hikikomori” is now globally common and is defined as “a pathological form of social segregation or isolation” [2]. It is essentially characterized by “physical isolation at home.” Kato et al. [2] outlined three criteria to fulfill the definition of hikikomori: (1) extreme social isolation at home; (2) a duration of isolation for six months or more; and (3) significant impairment or distress linked to (1). Community-dwelling persons often have mental illnesses, including schizophrenia, and suffer from illness-related conditions. A representative condition is “hikikomori”, or social withdrawal. It is difficult to distinguish the “hikikomori” from social withdrawal due to the symptoms of schizophrenia [2]. Furthermore, there are reports that approximately 30% of individuals in a hikikomori state are diagnosed with schizophrenia [3,4]. However, the prevalence of community-dwelling patients with schizophrenia who meet the criteria for hikikomori remains underreported. Therefore, it can be inferred that many individuals in the community exhibit symptoms of schizophrenia and hikikomori (SH).

In many countries, outreach methods have recently focused on caring for patients with mental illnesses. In Japan, psychiatric home-visit nursing (PHVN) services represent a cornerstone of outreach medical care for individuals residing within the community and grappling with mental disorders. The initiation of PHVN services occurs when a primary psychiatrist acknowledges the necessity of such services for their patient and subsequently issues an order for their provision [5]. Psychiatric home-visit nurses (PHVNs) visit the patient’s place of residence and assist them on the spot. This ensures that patients’ community life is maintained. PHVNs assume a pivotal role in this service by offering comprehensive support for patients, encompassing physical and mental health assessments, symptom management, psychological assistance, lifestyle guidance, and the empowerment of service users [6]. The scope of PHVNs’ support spans a wide spectrum, including nursing care tailored to mental disorder symptoms; the adjustment of daily routines, including sleep and nutrition; and fostering independence in everyday activities such as housekeeping, laundry, shopping, cooking, and financial management. Additionally, it entails the continued receipt of medical services such as outpatient care, daycare, and medication, as well as facilitating patients’ reintegration into society, including employment opportunities [5]. Setoya et al. [6] indicated lower admission rates and extended community stays among users benefiting from PHVN services.

The Ministry of Health, Labour and Welfare (MHLW) [7] reported that individuals with schizophrenia represent the largest cohort among those availing PHVN services within the realm of mental health. Considering that nearly 60% of individuals receiving PHVN services in Japan are patients with schizophrenia [7], the necessity of this service tailored to patients with SH becomes evident. Additionally, PHVNs have the potential to support individuals with SH in their pursuit of fulfilling community life via the delivery of PHVN services [8]. With the 2012 amendment to the medical insurance system in Japan, healthcare professionals providing PHVN services who did not have experience in psychiatric inpatient or outpatient settings were mandated to complete the training programs, which are conducted for approximately twenty hours [9]. Substantive instruction for nurses who have no experience in psychiatric nursing is left to each office providing PHVN services. This leads to PHVN practice that is only based on each nurse’s experience and intuition. Therefore, there is a pressing need to develop reasonable and effective PHVN practices tailored to individuals with mental illness.

The present study seeks to elucidate the overarching themes of the skilled PHVNs’ practices when caring for patients with SH, as they have not been clarified. This will provide a foothold for evidence-based practices for patients with SH. By uncovering these themes, PHVNs can provide support with conviction, even in situations where they may feel embarrassed or impatient during visits with patients dealing with SH. Such support will not only contribute to the maintenance of these patients’ community lives but also facilitate a sense of security and hope, enabling them to lead fulfilling lives within their community.

## 2. Materials and Methods

### 2.1. Design

To investigate the research question, this study employed a qualitative descriptive approach and conducted a thematic analysis. A qualitative descriptive approach is deemed appropriate in the absence of prior research [10]. This method entails a systematic process of identifying patterns within comprehensive descriptions, extracting and comparing prevalent themes in the practices of PHVNs when caring for patients with SH. It focuses on discerning similarities and differences in these practices. The present study adhered to the Consolidated Criteria for Reporting Qualitative Research (COREQ) checklist [11].

### 2.2. Sample and Recruitment

Convenience sampling was employed, drawing from home-visit nursing agencies offering PHVN services in Osaka Prefecture, which is located at the heart of Japan’s main island. It is the country’s second-largest city after Tokyo, with a population of approximately 8.8 million [12]. As of September 2022, there were 1751 home-visit nursing agencies operating in Osaka Prefecture [13]. Of these, approximately 1300 (73.7%) were home-visit nursing agencies providing PHVN services [14].

Between December 2020 and May 2021, the researcher explained the study’s outline to the managers of five home-visit nursing agencies via telephone. Multiple agencies were selected to minimize the influence of each agency’s culture and atmosphere on the study. Subsequently, the managers were approached to gauge their willingness to participate in the study. Following their agreement to participate, the managers were requested to recommend PHVNs who met the inclusion criteria for the study. Participant inclusion criteria comprised (a) PHVNs with a minimum of three years of experience [15], who were so-called skilled in providing PHVN services, and (b) PHVNs who had experience in delivering PHVN services to patients with SH. The introduced PHVNs were informed about the study’s purpose and methods, and their consent to participate was confirmed.

Upon obtaining consent, participants were instructed to envision a representative case of a patient diagnosed with schizophrenia who met the hikikomori criteria as defined by Kato et al. [2]. This definition encompasses marked social isolation within one’s home for a continuous period of at least six months, accompanied by significant functional impairment or distress. Furthermore, in alignment with Kato et al. [2], individuals who left their homes on four or more days per week were excluded from the study’s hikikomori criteria. Prior to the interviews, participants were asked to complete a questionnaire detailing their demographics and characteristics of the imagined case. No participant withdrew their consent to participate in the study either before or after the interview.

### 2.3. Data Collection

To collect data addressing the study objectives, one or two semi-structured interviews lasting between 20 and 80 min were conducted with each participant at a location and time of the participant’s choice. After reviewing the questionnaire responses, they were asked the following questions about the case: (1) What was your patient’s condition when you started? (2) How did you practice PHVN for the patient? (3) During your PHVN practice, did you implement any changes? If so, please tell us what practices you have changed, including those related to the patient’s condition. Participants were encouraged to freely discuss the details of the practices they employed. All interviews were conducted by a single researcher (M. M.), a female registered nurse holding a master’s degree with experience in delivering PHVN services, to ensure reliability. Interviews were audio recorded with the consent of the participants and transcribed verbatim by the same researcher (M. M.). Field notes and memos were generated immediately after the interview to capture nonverbal cues exhibited by the participants and the researcher’s reflections on the participants’ responses. The interview and analysis cycle were repeated for each case to obtain sufficient data.

### 2.4. Data Analysis

Data analysis followed Braun and Clarke’s [16] six-step inductive thematic analysis method. A detailed process of the analysis is presented below to contribute to transferability. Additionally, the participants’ own words were actively used in the analysis process. Initially, the researcher (M. M.) immersed herself in the data by repeatedly reviewing the verbatim transcripts while listening to the audio recordings. Furthermore, researchers (H. F. and T. H.) engaged in iterative readings of the transcripts to become well-acquainted with the data. Subsequently, the researcher (M. M.) devised a coding scheme regarding our research question in the PHVN practices from the verbatim transcripts and documented them. The three researchers (M. M., H. F. and T. H.) convened, discussed, and refined the coding schemes until a consensus was reached. The codes were then collated, and preliminary themes and subthemes were delineated. Upon completion of this analysis process for one case, the next interview and analysis were conducted. After analysis of all the cases, individual code extracts and the entire dataset were reviewed until a consensus was achieved among the three researchers regarding the final themes and subthemes. Nine overarching themes emerged from the data analysis and were named in accordance with their characteristics. The emerging themes, their meanings, and the analysis process were confirmed to ensure credibility by co-researchers, who comprised a qualitative researcher and a psychiatric nurse (M. H.), a qualitative researcher focused on people with hikikomori, and a psychiatric occupational therapist (K. Y.).

### 2.5. Ethics

This study received ethical approval from the Research Ethics Committee of the Graduate School of Health Sciences, Kobe University (approval number 971). Before data collection, all study participants were provided with a comprehensive explanation outlining the study’s purpose, content, and confidentiality. They were explicitly informed that participation was voluntary and that they could withdraw at any point. They demonstrated their capacity to provide informed consent and written consent was obtained from each one of them. No adverse events occurred during the interviews. To maintain the anonymity of participants, data were handled using identification numbers.

## 3. Results

Ten experienced PHVNs participated in the current study. Table 1 and Table 2 present the demographic details of the participants and the cases they discussed, respectively. Notably, all the cases discussed by the participants, hereafter referred to as patients, were diagnosed with schizophrenia and had been in a hikikomori condition for more than three years at the time of the survey.

Nine overarching themes emerged as fundamental to PHVNs’ practices. These themes are as follows: Understanding the patient’s world, Supporting the patients as they are, Providing a sense of relief, Having equal relationships, Exploring the right timing, Waiting for the appropriate timing, Working together on things, Continuing care for expanding the patient’s world, and Nursing care for the patient’s future (Table 3).

### 3.1. Theme: Understanding the Patient’s World

PHVNs’ practices were underpinned by the theme of *Understanding the patient’s world*. This theme encompassed their efforts to comprehend the patient via their words and actions. Furthermore, even if the patients’ situations were difficult to understand, the PHVNs’ practice of this theme signified their genuine interest in the experiential world of their patients. This theme was one of the starting points for creating appropriate PHVN practices for patients with SH. Within this theme, PHVNs were observed listening attentively to patients’ interests and desires, as well as engaging in meaningful dialogues to grasp their perspectives and thoughts.

*After all, he doesn’t really understand his unique expression. … So, of course, I also tried to understand what he was trying to say. But I just listened to what he liked and what he was worried about, and so on. … As we got to know each other, I understood that patients had a deep desire to contribute to society*.

(P7: participant7)

This theme was also evident in PHVNs’ practices of engaging in dialogues with the patient’s family, attentively listening to their perspectives, and gaining insights into the patient’s situation via the family’s narratives.

*When I was with his mother, I tried to carefully listen to her. … I often listened to her stories about the family. … I often heard that the patient was selfish because he was raised lovingly. As I listened to her stories, I think I got to know him better. I got to understand how he feels*.

(P4)

It was also manifested in PHVNs’ practices of candidly inquiring about the patient’s thoughts and emotions.

*It’s difficult (about every visit where the nurse is just silent and there). I wonder if it’s okay to keep it this way. …but the patient doesn’t refuse me. For now, he keeps asking me to just be there. Even so, I ask for his wishes every time I visit*.

(P6)

### 3.2. Theme: Supporting the Patients as They Are

The theme of *Supporting the patients as they are* was evident in the practices of PHVNs. This theme conveys that PHVNs respect the patient’s thoughts and tempo, accepting the patient’s hikikomori lifestyle as it exists. In particular, this theme is related to patients gaining the experience of being accepted as they are by PHVNs. It emerged from PHVNs’ practices of refraining from coercive attempts to reintegrate the patient into society.

*Well, the staff and I don’t think that it’s wrong that he doesn’t go out. I mean, he was like that. If he felt a great deal of distress for his shut-in, I would try to somehow get him out. But he was like this; it cannot be helped. Rather, if he goes out, he is going to be out of shape, he gets scared, and has diarrhea*.

(P5)

Additionally, this theme was reflected in PHVNs’ practice of respecting the patient’s refusal, even when suggesting an increase in the number of PHVNs to facilitate the accumulation of the patient’s interpersonal experiences.

*I asked him, “Can you accept a visit by other staff?” but he said, “I am nervous,” “I can’t talk about what we have been talking about,” and “I don’t want to talk to many people.” … Really, you know, it’s better he accepts various staff visits, but it’s not easy to obtain his agreement.*

(P9)

### 3.3. Theme: Providing a Sense of Relief

The theme of *Providing a sense of relief* was derived from the practices of PHVNs. This theme suggests that patients experience a sense of relief via their interactions with PHVNs and the services provided by them. In other words, this theme claims that PHVNs create their attitude not to deny or invade patients with SH in their interaction. It is exemplified by PHVNs’ practices of actively listening to the patient’s grievances without interjecting their own opinions and engaging in discussions on topics of interest to the patient.

*I tried to listen to the patient’s complaints, and I think I just nodded my head and listened to him until he finished all his complaints*.

(P2)

*He was very fond of talking about food and often asked me about it. He often asked me what kind of ramen I eat. We often talked about that kind of thing. I remember that, yes*.

(P4)

This theme was further underscored by PHVNs’ practices of exercising caution when providing or recommending nurse-led care.

*I tried to be careful not to force them or tell them to do something just because I was a nurse*.

(P3)

### 3.4. Theme: Having Equal Relationships

The theme of *Having equal relationships* permeated PHVNs’ practices. This theme underscores the notion that PHVNs should regard patients as fellows sharing the same timeframe and should establish relationships grounded in mutual respect. It emerged from PHVNs’ practices of actively engaging with the patient, sharing their aspirations, and collaborating closely.

*It’s one on one. He confided to me, “I want to become more energetic.” (omission), and we’ve been working on it together. So, we’re fellows*.

(P6)

This theme also emanated from PHVNs’ practices of vigilantly observing and honoring the patient’s choices, even when those choices diverged from the recommendations made by the PHVNs.

*She required my opinion, “A social worker told me to try living alone, but what do you think?” She also consulted with others and thought about it, but she didn’t decide to try living alone*.

(P10)

### 3.5. Theme: Exploring the Right Timing

The theme of *Exploring the right timing* was another prevailing theme in PHVNs’ practices. This theme suggests that nurses judiciously and proactively time their deliberate engagements with patients. This theme helps patients avoid being overloaded and distressed while interacting with PHVNs. It was discerned from PHVNs’ practices of introducing challenging subjects within the context of routine conversations with patients.

*So, while watching the game and having an ordinary conversation with the patient, I sometimes feel that if I ask this question now, he might answer. For the time being, I think he is more frank when watching or playing a game than when talking face to face*.

(P3)

This theme was also evident in PHVNs’ practices when assessing the patient’s readiness for the introduction of new services and long-term lifestyle changes, particularly those considered essential.

*She is hesitant about living alone. She makes excuses and hesitates about living alone. But, yeah, I’d like to continue to have her goal of living alone. I don’t want to give up yet. Yes. I’ll keep watching over her. If there is a time when it is possible (omission), I’d like her to experience living alone, even if it is a short experience, and learn how expensive and how hard it is*.

(P10)

It also arose from the practice of discerning cues that could lead to the development of novel care approaches while providing care to patients.

*Suddenly then (when I was wiping the body of a patient who did not want to be touched) (omission), I felt some response. While I was doing it, I thought it could be possible (to expand the range of wiping the patient’s body)*.

(P2)

### 3.6. Theme: Waiting for the Appropriate Timing

The theme of *Waiting for the appropriate timing* was interwoven into PHVNs’ practices. This theme suggests that PHVNs may recognize the necessity of introducing services and care to enhance the patient’s quality of life but exhibit caution in taking immediate action. PHVN explores the most likely timing for changes in the patient’s daily life and the care being provided based on the patient’s understanding and wishes. This approach was aligned with PHVNs’ practices that empathized with the patient’s emotions and refrained from coercive measures.

*Well, helper, guides, helper. He doesn’t think he needs them now. He doesn’t think of them at all. He thinks he can do it. In fact, he has the ability to do it. He thinks he can do it when the time comes, so I don’t recommend it now*.

(P2)

Furthermore, this theme was also exemplified by intentionally not providing PHVN care based on the assessment of the patient’s condition.

*Well, you know, there are days when he says no. I mean, you know, it’s really difficult (to go for a walk with). In terms of frequency, it depends on the season, but … sometimes he can go three times a month, and some months he can’t go at all*.

(P5)

This theme was also evident in the practices of having faith in the patients’ capabilities, watching over their own choices, and taking action.

*She’s strong. She has strength. … I know she worked very hard (at the community workshop). I really want her to show it. I think it’s a waste*.

(P10)

### 3.7. Theme: Working Together on Things

PHVNs’ practices were guided by the theme of *Working together on things*. This theme signifies that PHVNs proactively suggest collaborative efforts on activities when patients express interests and desires but do not take action to gradually realize these goals. It emerged from PHVNs’ practice of facilitating the alignment of the patient’s expressed wishes toward actualization and diligently evaluating even the slightest progress.

*He really liked coffee. The first time he was able to go out for a long time, he went to a cafe, which was in front of the station. He wanted me to take him there. Also, he wanted me to take his mother with him. … But in the end, he didn’t go up to the cafe and waited for us in the car at the parking area*.

(P4)

This theme was also complemented by the practices of jointly contemplating and endeavoring to address the patient’s anxieties.

*Kids, well, you know, kindergarten kids walking around. If they cry, he says, “It’s my fault.” He will say, “It’s my fault after all.” He falls into the feeling everything is like that. But I tell him he is okay because he’s with me, and I take him out for a walk*.

(P9)

Additionally, it was accompanied by the practice of mutual recognition of the results obtained as a result of working together to fulfill patients’ wishes.

*Really. But from there (omission), we found out that he wanted to regain his energy. Then, I said, “Exercise therapy is the only way.” The staff and I continued to work on his exercise. … He said, “Look, look!” He was able to sit up in a long sitting position! He was so happy and said, “Look at this!”*

(P6)

### 3.8. Theme: Continuing Care for Expanding the Patient’s World

The practices of PHVNs were underpinned by the theme of *Continuing care for expanding the patient’s world*. This theme suggests that PHVNs continue to work toward broadening the perspectives of patients who live with limited thinking, thereby enriching their thoughts and experiences. It involves conveying an objective perspective on the patient’s behavior and contrasting it with their self-perception. This theme emerged from PHVNs’ practices of helping patients understand the disparity between their self-image and objective reality.

*Yes, it is. I was rather frank and straightforward in telling him that he could not do well himself and that the people around saw him as not doing well. But I wonder if he listened to the voices around him or understood them. … Actually, he could not take any action. He had difficulties noticing his situation from an objective viewpoint*.

(P2)

Additionally, the theme emerged from PHVNs’ practices of encouraging patients to venture outside, motivating them to engage in outdoor activities, and redirecting the patient’s focus outward, often using their interests and a lack of physical activity as catalysts.

*Well, I tried to take him out from time to time. I also asked him where we could go for a walk*.

(P4)

*A few days ago, we went to a nearby park for the first time in a year...and took a short walk for about 10 min. I invited him to go outside and get some fresh air once in a while. He was gaining weight and wasn’t moving as much. He was also losing muscle strength, so I encouraged him to go for walks*.

(P9)

This theme was also complemented by PHVNs’ practices of providing explanations and demonstrating procedures while performing tasks that patients were unable to perform themselves.

*Medical tickets… The patient had to call the local government office in their residence and have it issued. So, we practiced because he couldn’t make a phone call in the first place*.

(P6)

### 3.9. Theme: Nursing Care for the Patient’s Future

PHVNs’ practices were guided by the theme of *Nursing care for the patient’s future*. This theme suggests the anticipation of potential future events in the patient’s life and the gradual provision of information and preparation of care delivery for those events. It encompasses practices such as discussing life after the death of a patient’s parents.

*Well, you know, his father and mother, when they die, he has to somehow earn a daily living on his own. I think it is important to provide information, show options, and have discussions in preparation for that time. He says he doesn’t want to live in an institution. But now he can choose various kinds of institutions, such as group homes (I will try to tell him)*.

(P5)

This theme also surfaced from PHVNs’ practices of envisioning the patient’s future life and establishing connections with places that could benefit the patient. PHVNs also strive to facilitate visits involving various PHVN staff and to arrange visits, including places aside from the patient’s home.

*About three or four years ago, the staff of the social welfare corporation in the town (where the patient lives) started part-time work on Wednesdays at our workplace. So, only on Wednesdays this staff began to accompany its visits. Well, I thought it would be good if it would lead to his participation in the welfare workshop, so I did it*.

(P4)

*In order not to encourage withdrawal... I was thinking about how to take him out, how to meet him outside his home. I was thinking about not completing the nursing care in his home. … So, in addition to visiting his house, I also used different locations, such as meeting him at Y Coffee Salon*.

(P1)

Additionally, this theme was complemented by PHVNs’ practices of offering information grounded in the observation of daily life and fostering the patient’s awareness, thereby empowering them to enhance their daily life and prepare for future challenges.

*The rhythm of his condition is also linked with the rhythm of his daily life. I often told him that I find him in a bad condition when he’s living an irregular life with his days and nights reversed*.

(P2)

## 4. Discussion

In the context of PHVNs’ practices for patients with SH, nine distinct themes emerged. Four themes, namely *Understanding the patient’s world*, *Supporting the patients as they are*, *Providing a sense of relief*, and *Having equal relationships*, were closely tied to PHVNs’ approach in a manner that respected the patient’s unique way of being while fostering and maintaining positive relationships. Additionally, despite the potentially high cost associated with home visits and medical services, the themes of *Waiting for the appropriate timing* and *Exploring the right timing* were notable in PHVNs’ practices. The remaining three themes, *Working together on things*, *Continuing care for expanding the patient’s world*, and *Nursing care for the patient’s future*, underscored the characteristic step-by-step collaboration between patients and PHVNs.

The four themes of *Understanding the patient’s world*, *Supporting the patients as they are*, *Providing a sense of relief*, and *Having equal relationships* primarily pertained to PHVNs’ approach. Notably, the first three themes bore a resemblance to key elements of psychiatric nursing practice when establishing relationships between patients and nurses [17]. Patients with SH, the focus of this study, often have limited external contact and interaction, which can be attributed to their coping strategies in managing stressors, including social withdrawal and a lack of spontaneity [2,18]. The provision of PHVN services is contingent upon a voluntary agreement between the patient and the home-visit nursing agency. Even when a psychiatrist deems the service necessary, and family members are supportive [19], most patients may still be hesitant to engage fully with the service. Therefore, these three themes underscore the importance of PHVNs respecting the current state of patients with SH and forging relationships between PHVNs and patients. Moreover, the theme of *Providing a sense of relief* emphasizes the importance of PHVNs in creating a safe and non-threatening space for patients to engage with others, given the limited or non-existent interactions experienced by these patients [20,21,22]. Via PHVNs’ practices aligned with these themes, individuals with SH may begin to experience social interactions, which can serve as a foundation for establishing connections with others.

The themes of *Exploring the right timing* and *Waiting for the appropriate timing* signify the nurses’ anticipation of proactive patient engagement. The two themes also encourage the nurses to dedicate themselves to exploring and waiting without trying to forcefully change the patients’ conditions [21,22]. In situations where the goal is to foster or maintain patient relationships or effect changes in the patient’s life, PHVNs intentionally refrain from taking action before the patient with SH initiates it. In essence, these themes reflect PHVNs’ strong belief in honoring the patient’s ability to make choices and act autonomously. Notably, these themes were not extensively reported in a previous study investigating PHVN practices broadly [23]. They may therefore be specific themes associated with the practice for patients with SH.

The themes of *Working together on things*, *Continuing care for expanding the patient’s world*, and *Nursing care for the patient’s future* signify PHVNs’ intentional and proactive involvement in the patients’ lives while concurrently building and maintaining relationships. Although these three themes might seem to conflict with the themes of *Waiting for the appropriate timing* and *Exploring the right timing*, they share commonalities in maintaining pace with the patient and respecting the patient’s choices. Patients with hikikomori tend to avoid situations involving interactions with others and environments with many people [2]. The theme of *Working together on things* may empower patients with SH to believe that they can work together on things with a nurse, potentially leading them to engage with others and venture into environments with increased social interaction. Via the theme of *Continuing care for expanding the patient’s world*, PHVNs strive to broaden the patients’ experiential world based on their individual interests and concerns. These practices are distinct from the support for social participation among individuals with hikikomori reported by Funakoshi et al. [24]. PHVNs’ practices for patients with SH may therefore serve as a means of enriching the patients’ realm of experiences to the fullest extent possible. Finally, the theme of *Nursing care for the patient’s future* underscores PHVNs’ efforts to prepare patients for future life events despite the impact of their condition. PHVNs’ practices in this context are forward-looking and consider the patients’ long-term well-being. Given the prolonged period of hikikomori experienced by patients in this study, PHVN services should be available over an extended duration. Consequently, the accumulation of these two types of involvement is crucial in broadening the world of patients with SH and equipping them to lead meaningful lives in the community in the future.

### Strengths and Limitations

This study demonstrated that PHVNs actively engaged in the application of nine distinct themes when caring for patients with SH. This is the first study to explore this practice for patients with SH, employing an exploratory research method due to the previously unknown nature of this phenomenon. Given the nature of the methodology, data saturation was not the aim. Currently, PHVN practices rely on the experiences of individual nurses, but we believe that this study’s results, along with additional research, will contribute to evidence-based practices in the future. Furthermore, combining research on the experiences of patients with SH and their families could aid further development of nursing practices for this patient group. Further research into PHVNs’ implementation of the themes obtained in this study holds the potential to yield more effective strategies in supporting the recovery of patients with SH.

Limitations of the study include the small sample size, using the convenience sampling method to recruit participants, and data collection from only one city. Therefore, the data obtained may be biased. Hence, future studies with a larger sample size are required to verify whether PHVNs’ practices are in line with the themes obtained in this study.

## 5. Conclusions

In the context of patients with SH, PHVNs employed practices characterized by nine distinct themes. Within the themes of *Understanding the patient’s world*, *Supporting the patients as they are*, *Providing a sense of relief*, and *Having equal relationships*, PHVNs prioritized the patients’ sense of relief and dedicated themselves to building and sustaining relationships while gaining insight into the patients’ unique perspectives. In the themes of *Exploring the right timing* and *Waiting for the appropriate timing*, PHVNs demonstrated trust in the patients’ capabilities and respected their individual pace, choosing to wait for the most opportune moments. Finally, in the themes of *Working together on things*, *Continuing care for expanding the patient’s world*, and *Nursing care for the patient’s future*, PHVNs collaborated with patients in a step-by-step manner, addressing even the smallest of concerns and fostering the development of the patients’ independent behavior.

This study revealed that PHVNs are pivotal in supporting patients with SH to sustain community living while experiencing a sense of relief and to lead meaningful lives. This is achieved via PHVNs’ diligent adherence to these thematic practices.

The results of this study will promote a shift from current practices, which depend on the experience and intuition of PHVNs, to more evidence-based practices in the future for patients with SH. Additionally, we believe that our findings, which emphasize appropriate approach and timing with patients and step-by-step collaboration, will significantly improve the practices of professionals who interact with patients with SH and may contribute to their recovery worldwide.

## Figures and Tables

**Table 1:** Demographics of participants (N = 10).

Item	Classification	N	%
Sex	Male	4	40
	Female	6	60
Age (years)	20–29	1	10
	30–39	1	10
	40–49	2	20
	50–59	6	60
Experience in PHVN (years)	3–5	2	20
	6–10	1	10
	11–15	7	70
Experience of working with patients with SH (cases)	1–5	2	20
	6–10	4	40
	11<	4	40
Length of time in charge of patients with SH (years)	1–3	3	30
	4–7	3	30
	8<	4	40

Abbreviations: PHVN: psychiatric home-visit nursing, SH: schizophrenia and hikikomori.

**Table 2:** Demographics of patients (N = 10).

Item	Classification	N	%
Main diagnosis	Schizophrenia	10	100
Sex	Male	9	90
	Female	1	10
Age (years)	30–39	2	20
	40–49	4	40
	50–59	2	20
	60–69	2	20
Living alone	Yes	3	30
	No	7	70
Employment experience	Yes	5	50
	No	5	50
Marriage status	No	10	100
Duration of hikikomori (years)	3–6	4	40
	7–10	1	10
	11–20	4	40
	21<	1	10
Frequency of leaving home	0 time	3	30
	1–2 times per week	2	20
	1–3 times per month	5	50
GAF score at the beginning of PHVN service	1–20	1	10
	21–30	1	10
	31–40	4	40
	41–50	4	40
Number of times receiving PHVN care	1 time per week	5	50
	2 times per week	2	20
	3 times per week	3	30

Abbreviations: PHVN: psychiatric home-visit nursing, GAF: global assessment of functioning.

**Table 3:** Theme titles.

Theme Titles
Understanding the patient’s world
Supporting the patients as they are
Providing a sense of relief
Having equal relationships
Exploring the right timing
Waiting for the appropriate timing
Working together on things
Continuing care for expanding the patient’s world
Nursing care for the patient’s future

## Data Availability

No new data were created or analyzed in this study. Data sharing is not applicable to this article.
